# A Case of QT Prolongation Associated with Panhypopituitarism

**DOI:** 10.1155/2013/989745

**Published:** 2013-05-09

**Authors:** Dilek Arpaci, Mustafa Volkan Demir, Tayfun Garip, Ali Tamer

**Affiliations:** Department of Internal Medicine, Sakarya Education and Research Hospital, 54290 Sakarya, Turkey

## Abstract

We describe a 37-year-old patient with panhypopituitarism who experienced symptoms and signs of hormonal insufficiency and QT prolongation on electrocardiogram without electrolyte disturbances. After hormonal (steroidal and thyroid) replacement therapy electrocardiographic findings were normalized. Hormonal disorders should be considered as a cause of long QT intervals which may lead to torsade de pointes, even if plasma electrolyte levels are normal, because life-threatening arrhythmia is treatable by supplementation of the hormone that is lacking.

## 1. Introduction

QT prolongation has various causes, including drug toxicity, electrolyte abnormality, myocarditis, cerebrovascular disease, chromosomal abnormalities of cardiac ion channels, and hormonal disorders such as hypopituitarism, hypothyroidism, and adrenal insufficiency [1–5]. It is well known that QT prolongation can be associated with polymorphic ventricular tachycardia (VT), which is usually resistant to antiarrhythmic drug therapy.

 We experienced a case of long QT intervals associated with panhypopituitarism that developed idiopathically as a result of empty sella.

## 2. Case Report 

 A 37-year-old woman was admitted to our hospital because of amenorrhea and infertility. She also suffered from fatigue, numbness, and swelling in hands and foot. Her systolic blood pressure was found to be <90 mmHg. She had mild myxedema without acromegalic phenotype. No galactorrhea was described. Her past history was remarkable. She had a traffic accident at 14 years of age. She had regular menstrual cycles until 20 years of age. After 20 years of age, no menstrual bleedings have yet occurred nowadays. She also complained from mild headache around eyes. Polydipsia and polyuria were not defined. 

**Figure 1:**
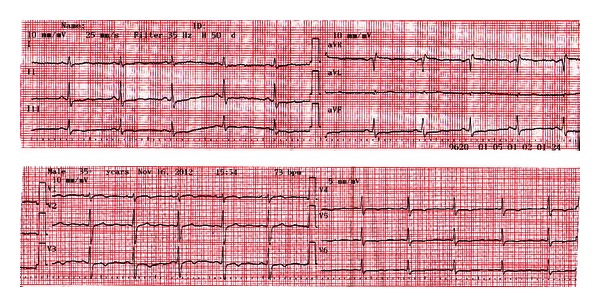
Twelve-lead electrocardiogram on admission shows long QT intervals and inverted T waves.

 Laboratory tests include serum biochemistry, and hormones revealed glucose: 86 mg/dL (70–105 mg/dL), urea: 17.1 mg/dL (18–45 mg/dL), creatinine: 0.7 mg/dL (0.57–1.11 mg/dL), calcium: 8.8 mg/dL (8.4–10.2 mg/dL), phosphorus: 3.3 mg/dL (2.5–4.5 mg/dL), magnesium: 2.1 mg/dL (1.7–3.1 mg/dL) Na: 141 mmol/L (136–145 mmol/L), K: 4.7 mmol/L (3.5–5.1 mmol/L), fT3: 2.12 pmol/L (2.63–5.70 pmol/L), fT4: 5.81 pmol/L (9.01–19.04 pmol/L), TSH: 1.28 IU/mL (0.35–4.94 IU/mL), LH: 0.02 mIU/mL, FSH: 0.06 mIU/mL, PRL: 1.81 ng/mL (5.18–26.53 ng/mL), oestrogen: 24 pg/mL, progesterone: 0.1 ng/mL, cortisol: 0.01 *μ*g/dL (3–19 *μ*g/dL), and ACTH: 10.9 pg/mL (22.5–95.3 pg/mL). She was hypotensive without any electrolyte imbalance. We administered stress-dose steroid and then reduced to replacement dose by titration. At followup, hypotension was improved. Thyroid function tests displayed lower fT3 and fT4 and normal TSH levels which was thought to be secondary hypothyroidism. We gave her levothyroxine (LT4) replacement. Urine-specific gravity was 1011 and fluid intake and urine output were normal. She had no menstrual bleedings for seventeen years, and also serum progesterone level, was low so called hypogonadism. She was consulted with gynecologist and endometrial wall thickness was lower, so she was offered to take estrogen progesterone replacement treatment. With all these findings, we suspected panhypopituitarism. Hypophyseal magnetic resonance imaging (MRI) was taken and was shown as hypophyseal hypoplasia with dimensions of 4.6 mm height and 8.5 mm width. And also posterior pituitary bright spot was seen on MRI. Twelve-lead electrocardiogram was taken (Figure 1).

**Figure 2:**
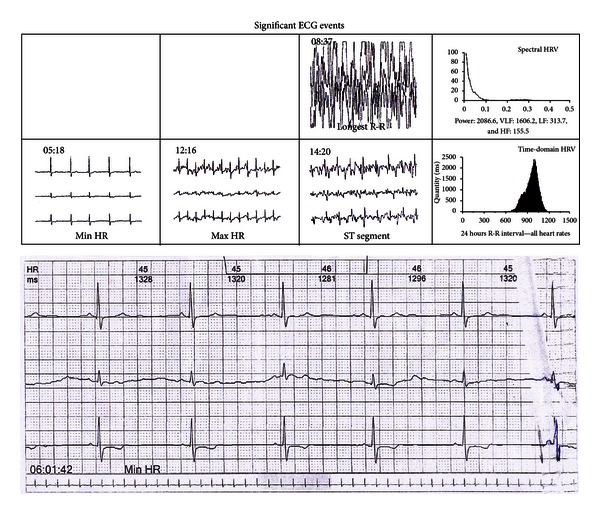
Some significant ECG events and minimal heart rate ECG record which were detected at 24-hour rythm holter ECG.

At cardiac auscultation murmur was heard. Patient was consulted with a cardiologist. A two-dimensional transthoracic echocardiography was taken it showed normal left ventricular systolic function with 65% ejection fraction, left atrium enlargement, and mild mitral and tricuspid regurgitation. A 24-hour rhythm holter electrocardiography (ECG) was taken (Figure 2); it showed that basic rhythm was sinusal rhythm, minimal heart rate was 44/min, mean heart rate was 62/min, maximal heart rate was 107/min, and no ventricular/supraventricular tachycardia or pause was detected. Maximal QT/QTc interval was 549 ms. 

**Figure 3:**
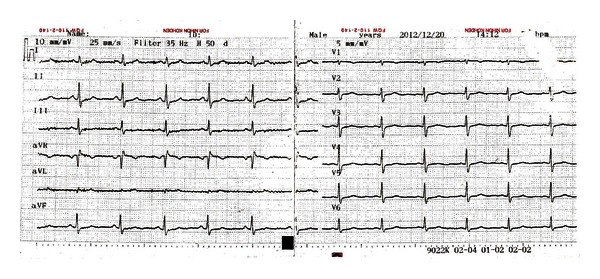
Four weeks after starting steroid and thyroid replacement therapy, twelve-lead electrocardiogram demonstrates normal QT intervals.

We considered that QT prolongation may be caused by hypopituitarism. Steroid replacement therapy was started on the 5th hospital day and thyroid replacement therapy on the 11th hospital day. Three weeks after starting steroid replacement therapy, the levels of cortisol and thyroid hormone were normalized. Two-dimensional echocardiography showed normal cardiac wall and valve motions. Also, an ECG showed QT normalization (Figure 3). QT/QTC interval was measured 364/392 ms.

## 3. Discussion 

 We described a case of QT prolongation associated with anterior hypopituitarism without plasma electrolyte abnormality. The cause of hypopituitarism could not be defined in our patient. QT prolongation may result from electrolyte disturbances, the use of various antiarrhythmic drugs, phenothiazines or tricyclic antidepressants, liquid protein diets, intracranial events, bradyarrhythmias, and hormonal disorders. In our patient, no drug use was described what mentioned above. But hormonal disorders (hypocortisolemia and hypothyroidism) were found in our patient.

 QT prolongation may lead to torsade de pointes a form of polymorphic ventricular tachycardia which can cause sudden cardiac death. In the literature, there is only one report that described a case of an association between torsade pointes and hypopituitarism [6].

 Electrocardiographic abnormalities commonly associated with hypopituitarism are low QRS voltage, ST-segment depression, inverted T waves, and a prolonged QT interval [7, 8]. Although the mechanism remains unclear, glucocorticoid deficiency, an intracellular-extracellular electrolyte imbalance of myocytes, and histopathological changes in the myocardium are thought to play a role in this disorder. Recently, it was reported that glucocorticoids upregulate Kv 1.5 K^+^ channel gene expression in the rat ventricle [9]. Iga et al. [10] suggested that catecholamine release induced by hypoglycemia might cause arrhythmia or abnormal wall motion of the left ventricle in patients with adrenal insufficiency. In our patient, QT prolongation without any arrhythmias occurred in the absence of hypoglycemia or plasma electrolyte abnormalities. Some previous reports [4, 11] have suggested that hypomagnesemia induced by adrenal insufficiency might cause myocytic intracellular-extracellular electrolytic imbalance, resulting in shortening of the effective refractory period and prolongation of the relative refractory period. In our patient, serum magnesium level was normal. Inverted T waves and prolonged QT intervals seen in our patient might be mediated by a hormonal modulation of ion channels of cardiac cells, which could contribute to QT prolongation and lead to polymorphic ventricular tachycardia before plasma electrolyte abnormality. But in our patient, polymorphic ventricular tachycardia did not occur. We found lower serum thyroid hormone levels and plasma cortisol levels. After steroid and thyroid hormone replacement therapy, the QT interval was normalized and no further VT developed.

 In conclusion, hormonal disorders must be examined as a cause of polymorphic ventricular tachycardia associated with long QT intervals even if plasma electrolyte levels are normal, because supplementation of insufficient hormone may permanently cure life-threatening arrhythmia.
